# A bibliometric profile of optogenetics: quantitative and qualitative analyses

**DOI:** 10.3389/fnins.2023.1221316

**Published:** 2023-06-22

**Authors:** Zhonghan Zhou, Xuesheng Wang, Xunhua Li, Limin Liao

**Affiliations:** ^1^Shandong University, Jinan, Shandong, China; ^2^Department of Urology, China Rehabilitation Research Center, Beijing, China; ^3^University of Health and Rehabilitation Sciences, Qingdao, Shandong, China; ^4^China Rehabilitation Science Institute, Beijing, China; ^5^Beijing Key Laboratory of Neural Injury and Rehabilitation, Beijing, China; ^6^School of Rehabilitation, Capital Medical University, Beijing, China

**Keywords:** optogenetics, bibliometrics, quantitative analysis, qualitative analysis, hot topic

## Abstract

**Introduction:**

Optogenetics is a rapidly developing field combining optics and genetics, with promising applications in neuroscience and beyond. However, there is currently a lack of bibliometric analyses examining publications in this area.

**Method:**

Publications on optogenetics were gathered from the Web of Science Core Collection Database. A quantitative analysis was conducted to gain insights into the annual scientific output, and distribution of authors, journals, subject categories, countries, and institutions. Additionally, qualitative analysis, such as co-occurrence network analysis, thematic analysis, and theme evolution, were performed to identify the main areas and trends of optogenetics articles.

**Results:**

A total of 6,824 publications were included for analysis. The number of articles has rapidly grown since 2010, with an annual growth rate of 52.82%. Deisseroth K, Boyden ES, and Hegemann P were the most prolific contributors to the field. The United States contributed the most articles (3,051 articles), followed by China (623 articles). A majority of optogenetics-related articles are published in high-quality journals, including NATURE, SCIENCE, and CELL. These articles mainly belong to four subjects: neurosciences, biochemistry and molecular biology, neuroimaging, and materials science. Co-occurrence keyword network analysis identified three clusters: optogenetic components and techniques, optogenetics and neural circuitry, optogenetics and disease.

**Conclusion:**

The results suggest that optogenetics research is flourishing, focusing on optogenetic techniques and their applications in neural circuitry exploration and disease intervention. Optogenetics is expected to remain a hot topic in various fields in the future.

## 1. Introduction

Optogenetics is a rapidly developing field combining optics and genetics to control cellular activity with high spatial and temporal resolution using light (Editorial, 2010). The procedures of optogenetics include directing the light-sensitive proteins to specific cells, delivering light to specific tissues, and measuring the resulting changes at the cellular, tissue, or organ level. In 2010, optogenetics was named “Method of the Year” by Nature Methods and has since attracted wide attention (Editorial, 2010). Overall, optogenetics has become a powerful tool for elucidating the mechanisms of neural circuitry and has promising applications in both basic and clinical research. Bibliometric analysis is a powerful tool for tracking research trends within a particular field (Wang et al., 2022). By statistical analysis, it can objectively identify research contributions from various countries, institutions, journals, and authors and provide insights into the direction of future research, such as hotspots and emerging issues. However, it is noted that no bibliometric analysis of optogenetics has been conducted, and limited attention has been given to predicting frontiers and research hotspots in this field. Therefore, we preformed this bibliometric analysis to get a better understanding of this emerging area.

## 2. Method

**Figure 1:**
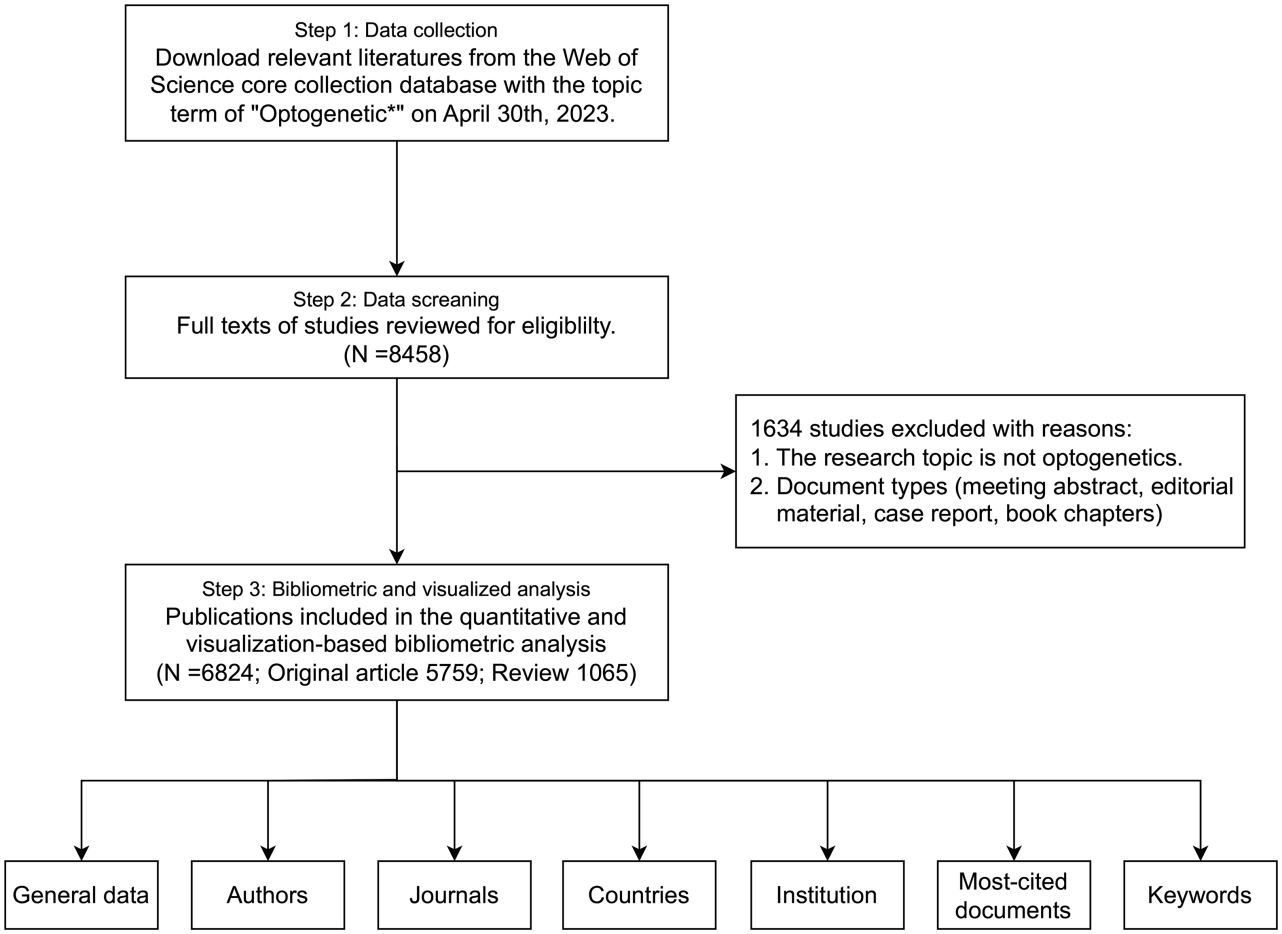
Flow chart of the bibliometric search on optogenetics.

We downloaded relevant literatures from the Web of Science Core Collection Database on April 30th, 2023 (Figure 1). “Optogenetic*” was the only topic term, and the period was set from 2002 to 2022. Language type was not restricted for the search. Two reviewers independently reviewed the titles and abstracts of the records. Any disagreements during the screening process were resolved through discussion or consulting a third reviewer if required. The online literature including both full documents and cited references was exported to plain text format and imported into R software (version 4.2.0). The R package bibliometrix was utilized to conduct an extensive analysis of the optogenetics literature (Aria and Cuccurullo, 2017). The quantitative analysis was performed to outline the annual scientific production and distribution of authors, journals, categories, countries, and institutions. A collaboration network was established to illustrate the cooperative relationships in this field. Furthermore, we performed qualitative analysis utilizing co-occurrence network analysis, thematic analysis, and theme evolution to identify the main areas and trends of optogenetics articles.

## 3. Results

### 3.1. General data

**Figure 2:**
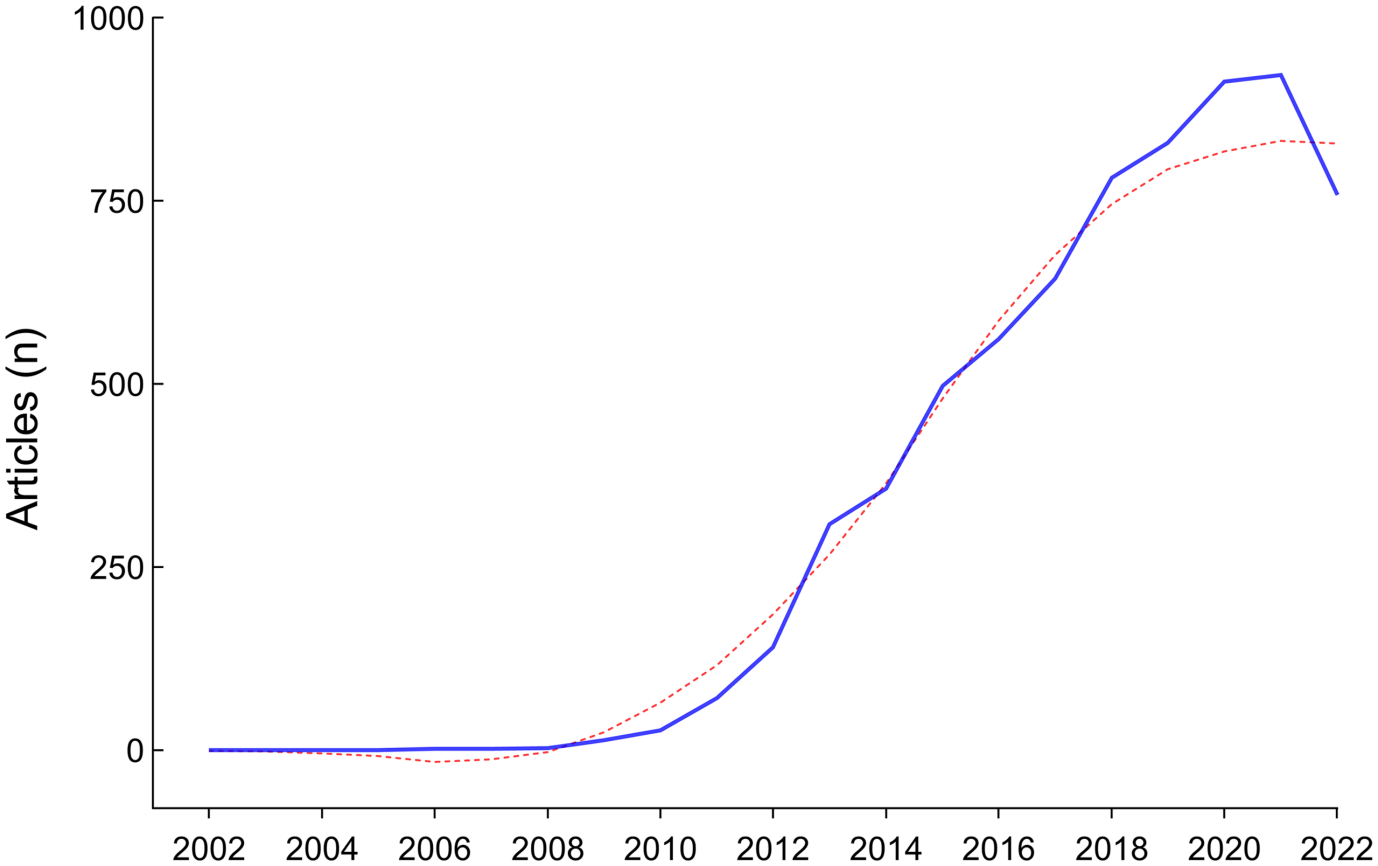
Annual scientific production from 2002 to 2022.

A total of 8,458 papers were collected from the WOS database (Figure 1). Of these, 1,634 papers, including book chapters, retracted publications, proceedings papers, editorial materials, and irrelevant papers were excluded. After exclusions, 6,824 publications, including 5,759 original research (84.39%) and 1,065 reviews (15.61%), remained for analysis. The number of articles has exhibited a rapid growth trend since 2010, with an annual growth rate of 52.82%, and the year 2021 reached a peak of 922 publications, indicating the field is a hotspot with explosive growth (Figure 2).

### 3.2. Distribution of authors

**Figure 3:**
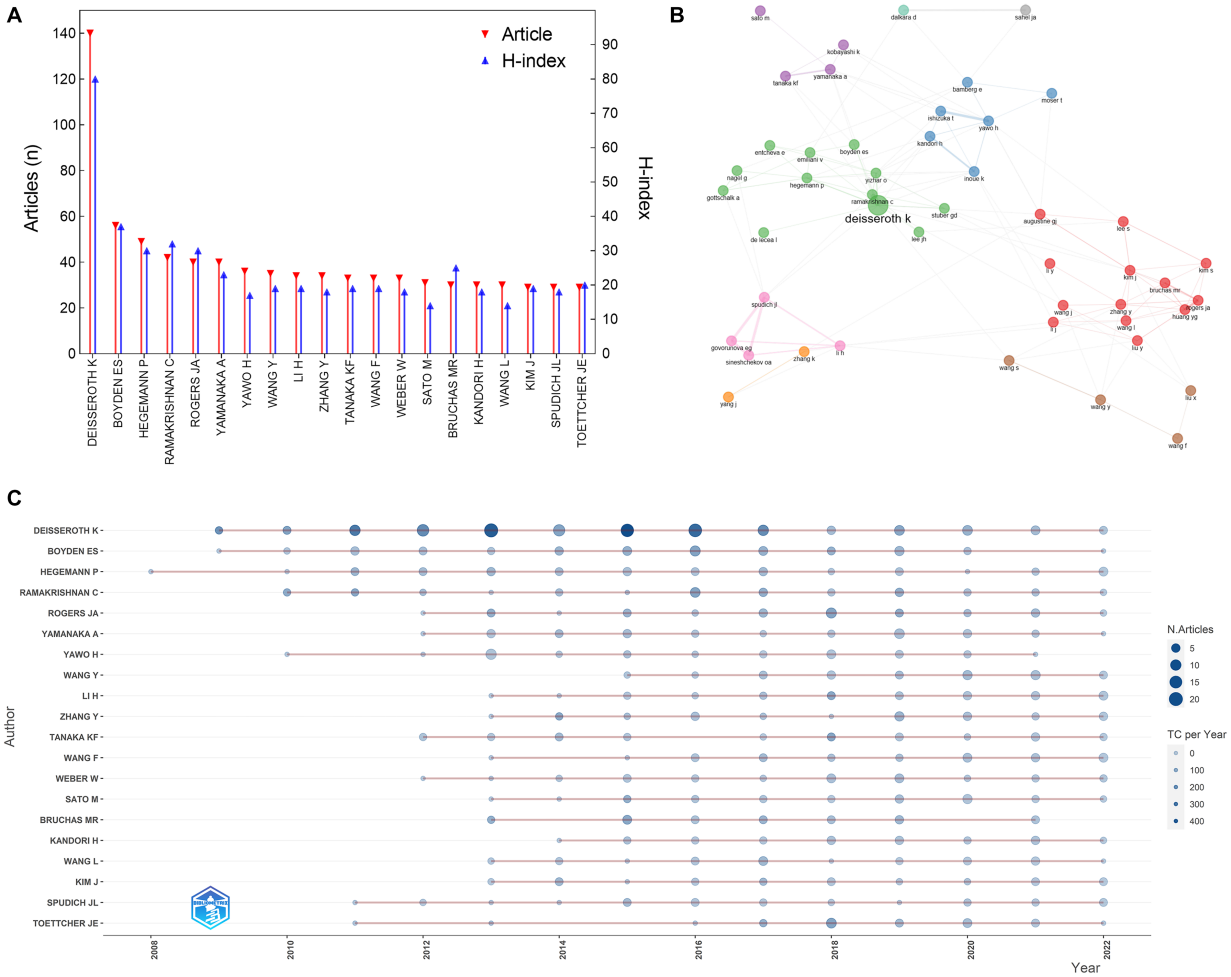
Distribution of authors in the field of optogenetics. **(A)** The top 20 most productive authors. **(B)** Map of the collaboration network analysis of the authors. **(C)** Publications of the top 20 authors over time.

We analyzed the distribution of authors to identify the top contributors to the field (Figure 3). Over 24,445 authors contributed to the 6,824 optogenetics-related studies. After some pioneers designed and implemented optogenetic approaches, a large number of researchers flooded into this field, especially after 2010. Deisseroth K had the highest publication count with 140 articles, followed by Boyden ES and Hegemann P with 56 and 49 articles, respectively. The Author’s Local Impact can be measured by the H-index, with Deisseroth K ranking first (H-index: 80), followed by Boyden ES (H-index: 37), Ramakrishnan C (H-index: 32), Hegemann P (H-index: 30), and Rogers JA (H-index: 30).

### 3.3. Distribution of journals and subject categories

**Figure 4:**
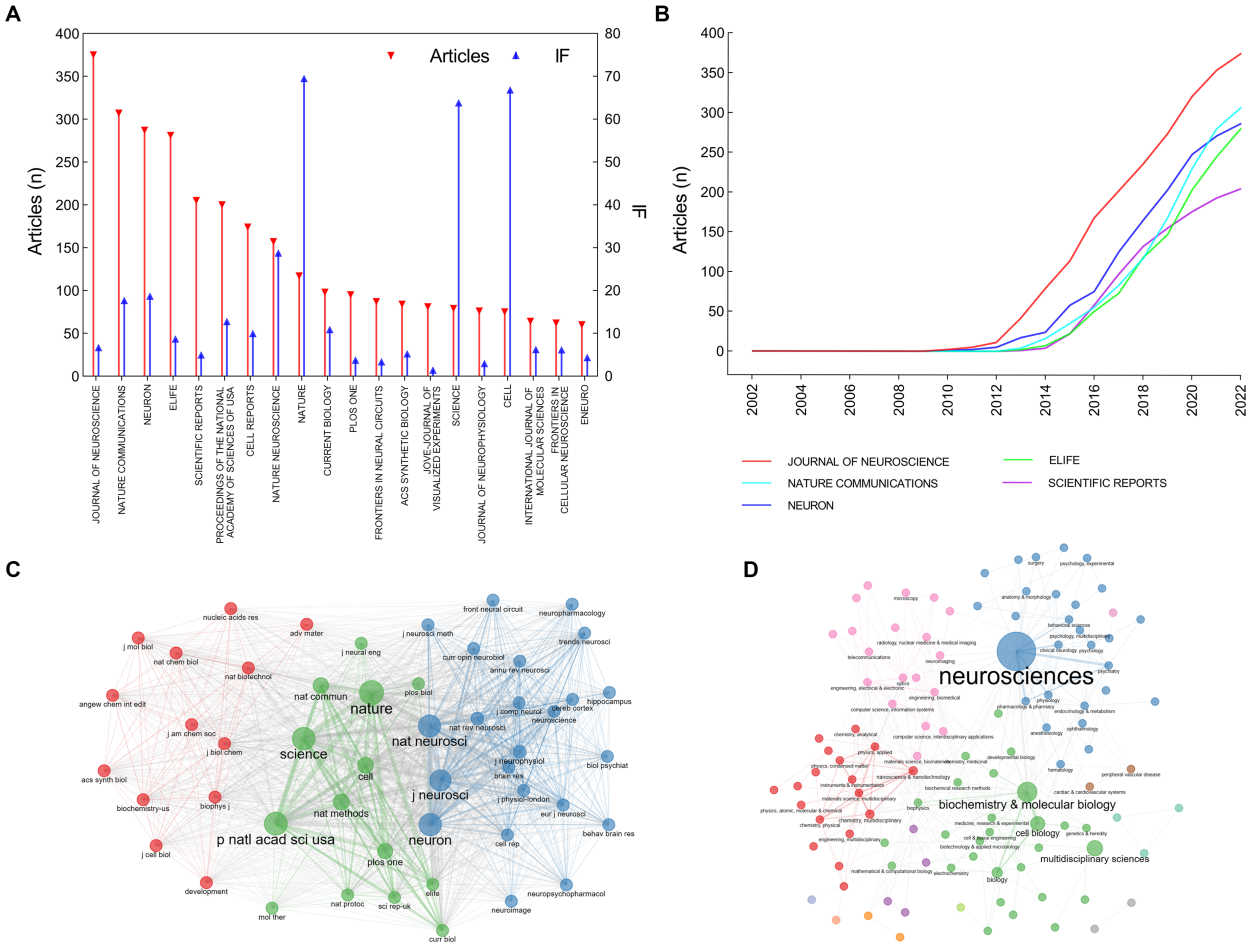
Distribution of journals and subject categories in the field of optogenetics. **(A)** The top 20 most relevant sources. **(B)** Publications of the top 5 sources over time. **(C)** Co-citation network of journals. **(D)** Co-occurrence network of subject categories.

The articles on optogenetics were published in more than 954 journals (Figure 4). JOURNAL OF NEUROSCIENCE published 375 articles, which accounted for 5.50% of all articles, followed by NATURE COMMUNICATIONS (307 articles, 4.50%), NEURON (287 articles, 4.10%), and ELIFE (281 articles, 4.16%), and SCIENTIFIC REPORT (205 articles, 3.00%). Research on optogenetics in these journals showed a rapid growth trend after 2012–2014. Journal impact factor (IF) and Journal Citation Reports (JCR) partition are important indicators measuring the academic impact of a journal and the quality of its publications. NATURE had the highest IF in 2021 (IF = 69.504, JCR Q1, 117 articles), followed by CELL (IF = 66.850, JCR Q1, 75 articles), SCIENCE (IF = 63.832, JCR Q1, 79 articles), NATURE NEUROSCIENCE (IF = 28.771, JCR Q1, 157 articles), and NEURON (IF = 18.688, JCR Q1, 287 articles). A majority of articles have been published in high-quality neuroscience or multidisciplinary life science journals. We conducted an analysis of the research categories and found that these articles mainly belong to four subjects: neurosciences (blue), biochemistry and molecular biology (green), neuroimaging (pink), and materials science/multidisciplinary (red).

### 3.4. Distribution of countries and institutions

**Figure 5:**
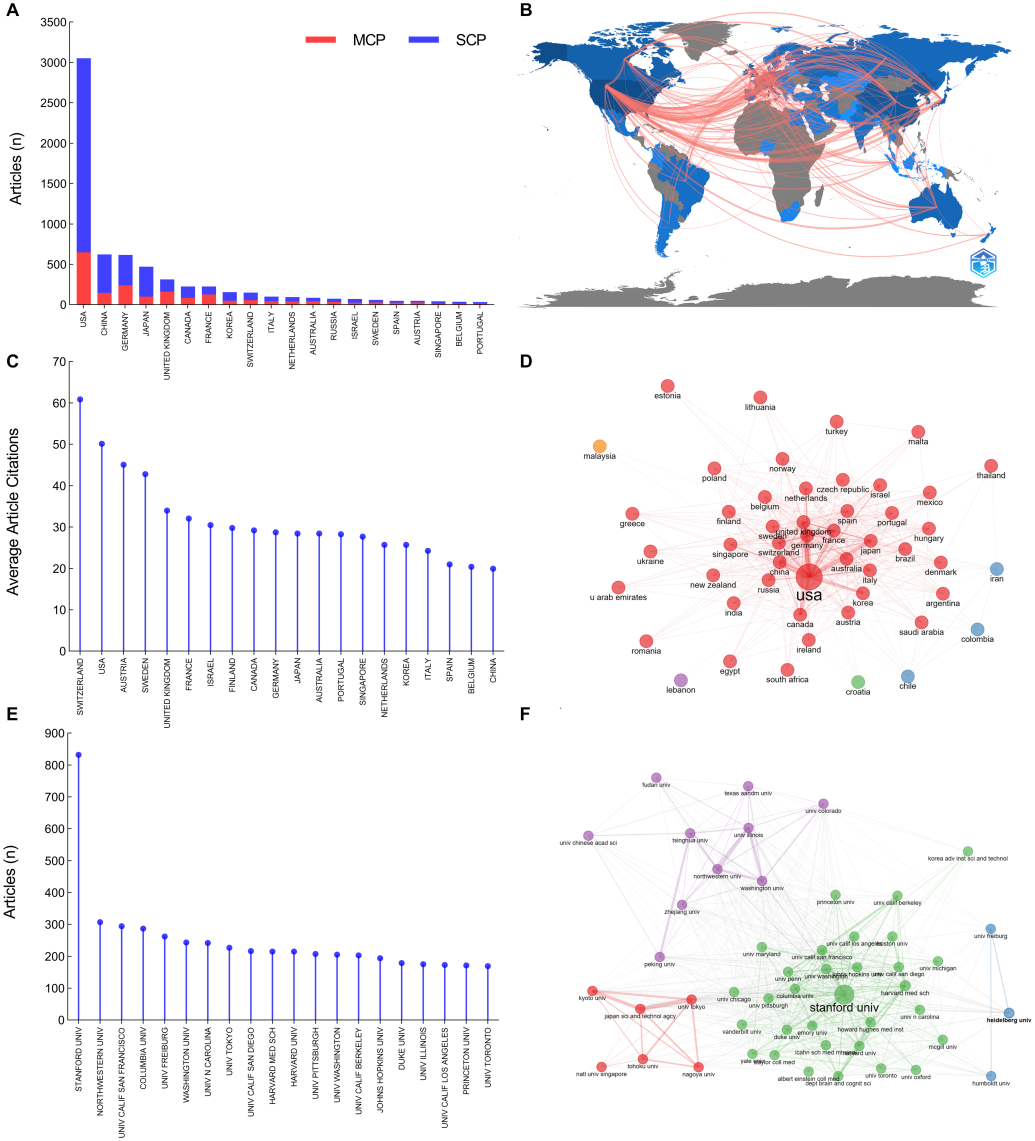
Distribution of countries and institutions in the field of optogenetics. **(A)** The top 20 most productive countries divided by single country publications (SCPs) and multiple country publications (MCPs). **(B)** Map of national collaborations. **(C)** The top 20 most influential countries ranked by average article citations. **(D)** Map of the collaboration network analysis of the countries. **(E)** The top 20 most productive institutions. **(F)** Map of the collaboration network analysis of the institutions.

The authors of the included publications were affiliated with 51 countries/regions (Figure 5). The United States was the country with the highest number of publications (3,051 articles, 44.71%), followed by China (623 articles, 9.13%), Germany (616 articles, 9.03%), Japan (470 articles, 6.89%), and United Kingdom (313 articles, 5.25%). There were 3,439 institutes involved in the field of optogenetics. Stanford University ranked first with 832 articles (12.19%), followed by Northwestern University (307 articles, 4.50%), the University of California, San Francisco (294 articles, 4.31%), Columbia University (287 articles, 4.21%), and University of Freiburg (262 articles, 3.84%). The dominance of the United States in optogenetics is undeniable, according to the number of publications and article citations. The percentage of international co-authorships is 30.01%, indicating a significant level of collaboration between researchers from different countries. Although China ranks second in publication output, its article citation rate ranks only 20th globally. Zhejiang University (167 articles, 2.45%) was the leading institution in China regarding the number of optogenetics articles and ranked 23rd globally.

### 3.5. Most cited documents and co-occurring keywords

**Figure 6:**
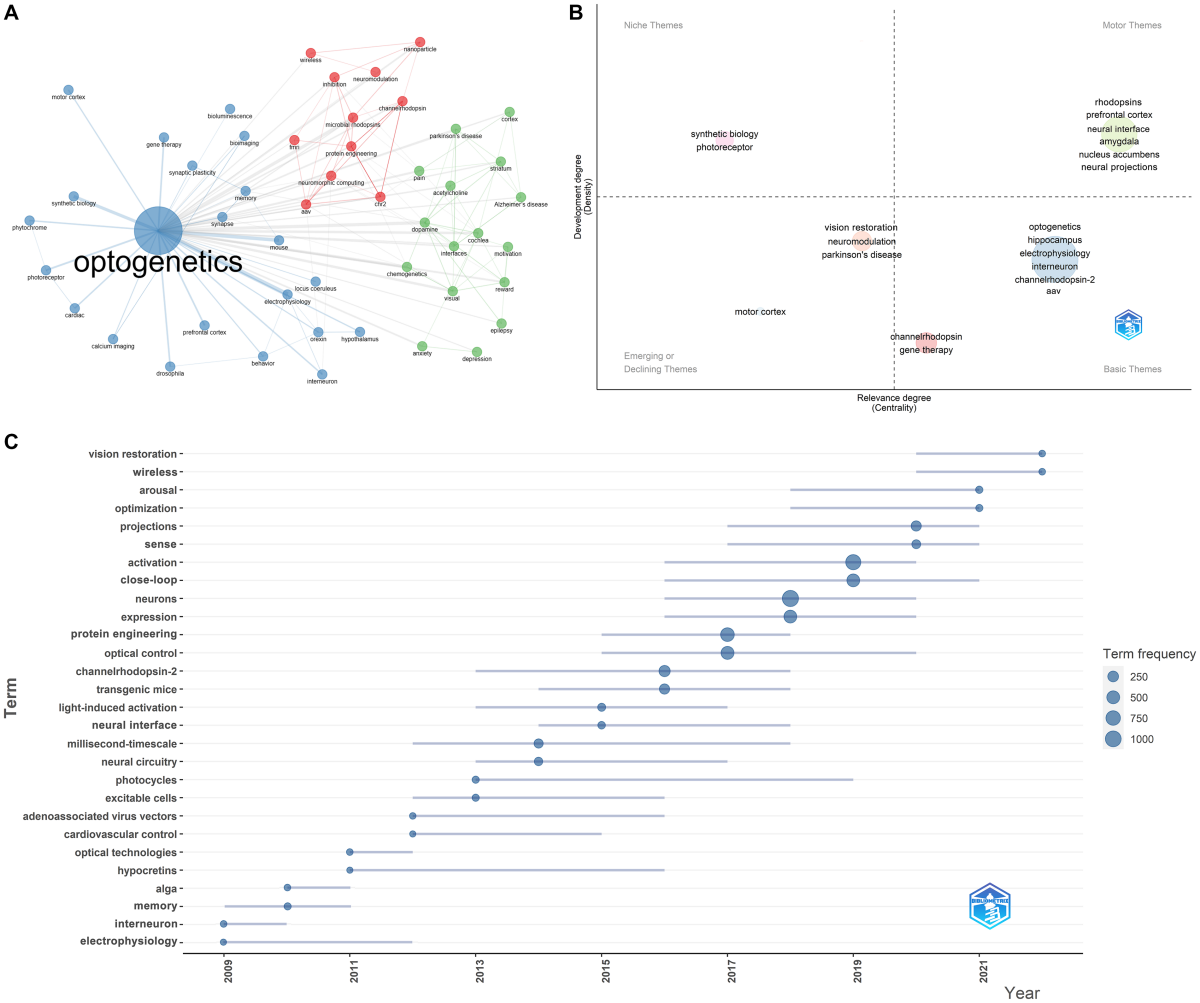
Most cited documents and co-occurring keywords in the field of optogenetics. **(A)** Map of the clustered co-occurrence network analysis based on the author’s keywords. **(B)** Thematic map analysis of author’s keywords. **(C)** Production of the keywords over time.

As shown in Table 1, the 20 most globally cited documents have been identified, and Deisseroth K accounts for 7 of them. All of them were published in high-quality journals, including NATURE and SCIENCE. The most cited document, “Parvalbumin neurons and gamma rhythms enhance cortical circuit performance” was published by Deisseroth K in NATURE in 2009 (Sohal et al., 2009). These 20 articles primarily focus on the following topics: improvements to optogenetic techniques, including channelrhodopsins functions, wireless light delivery devices, upconversion nanoparticle-mediated optogenetics, et al.; neural function and pathways, involving vision, anxiety, memory, et al.; optogenetics and diseases, including Parkinson’s disease, pain, depression, et al. These research topics reflect the forefront and hotspots worldwide. We have furtherly executed a co-occurrence network analysis and identified three clusters related to optogenetics research: optogenetic components and techniques (red), optogenetics and neural circuitry (blue), optogenetics and disease (green) (Figure 6). Thematic map analysis was conducted to demonstrate the development degree (density) and relevance degree (centrality) of the identified topics. This strategic diagram enabled the identification of the following categories: hot topics located in the upper-right quadrant (rhodopsins, neural interface, neural projections, et al.), basic topics in the lower-right quadrant (electrophysiology, interneuron, ChR2, AAV, et al.), niche topics in the upper-left quadrant (which have been strongly developed but still hold a marginal position in the domain under investigation, including synthetic biology, photoreceptor, et al.), and peripheral topics in the lower-left quadrant (which have not been fully developed, including vision restoration, neuromodulation, Parkinson’s disease, motor cortex, et al.). When taking the time dimension into the analysis, vision restoration, wireless, projections, sense, close-loop, protein engineering, et al., were top keywords in the past 5 years, while neural interface, neural circuitry, AAV, memory, interneuron, electrophysiology, et al., were hot topics 5 years ago.

**Table 1:** List of the 20 most global cited documents.

*n*	Year	CA	Journal	IF	Title	TC	TCPY	NTC
1	2009	Deisseroth K	NATURE	69.5	Parvalbumin neurons and gamma rhythms enhance cortical circuit performance	1704	113.60	6.16
2	2011	Deisseroth K	NAT METHODS	48.0	Optogenetics	1,237	95.15	10.48
3	2014	Boyden ES; Wong GKS	NAT METHODS	48.0	Independent optical excitation of distinct neural populations	1,134	113.40	15.00
4	2009	Deisseroth K	SCIENCE	63.7	Optical deconstruction of parkinsonian neural circuitry	1,102	73.47	3.99
5	2015	Lüthi A	NAT REV NEUROSCI	38.8	Neuronal circuits for fear and anxiety	900	100.00	12.33
6	2013	Bruchas MR; Rogers JA	SCIENCE	63.7	Injectable, cellular-scale optoelectronics with applications for wireless optogenetics	820	74.55	9.35
7	2011	Deisseroth K	NATURE	69.5	Amygdala circuitry mediating reversible and bidirectional control of anxiety	807	62.08	6.84
8	2013	Pfeffer CK; Scanziani M	NAT NEUROSCI	28.8	Inhibition of inhibition in visual cortex: the logic of connections between molecularly distinct interneurons	770	70.00	8.78
9	2013	Han MH	NATURE	69.5	Rapid regulation of depression-related behaviors by control of midbrain dopamine neurons	717	65.18	8.17
10	2015	Deisseroth K	NAT NEUROSCI	28.8	Optogenetics: 10 years of microbial opsins in neuroscience	696	77.33	9.54
11	2010	Deisseroth K	CELL	66.9	Molecular and cellular approaches for diversifying and extending optogenetics	675	48.21	4.20
12	2012	Gottschalk A; Mayer G; Heckel A	ANGEW CHEM INT EDIT	16.8	Light-controlled tools	666	55.50	8.42
13	2013	Tye KM; Deisseroth K	NATURE	69.5	Dopamine neurons modulate neural encoding and expression of depression-related behavior	661	60.09	7.54
14	2013	Kepecs A	NATURE	69.5	Cortical interneurons that specialize in disinhibitory control	657	59.73	7.49
15	2018	Chen S; Liu XG; McHugh TJ	SCIENCE	63.7	Near-infrared deep brain stimulation via upconversion nanoparticle-mediated optogenetics	644	107.33	15.96
16	2018	Bednarkiewic A; Liu XG; Jin DY	NAT COMMUN	17.7	Advances in highly doped upconversion nanoparticles	630	105.00	15.61
17	2014	Jonas P	SCIENCE	63.7	Interneurons. Fast-spiking, parvalbumin^+^ GABAergic interneurons: from cellular design to microcircuit function	620	62.00	8.20
18	2015	Ng TN; Bao ZN	SCIENCE	63.7	A skin-inspired organic digital mechanoreceptor	595	66.11	8.15
19	2015	Zeng HK	NEURON	18.7	Transgenic mice for intersectional targeting of neural sensors and effectors with high specificity and performance	587	65.22	8.04
20	2014	Malinow R	NATURE	69.5	Engineering a memory with LTD and LTP	582	58.20	7.70

CA, corresponding author; IF, impact factor; TC, total citations; TCPY, total citations per year; NTC, normalized total citations.

## 4. Discussion

This bibliometric analysis explored optogenetics-related literature over the past 20 years (2002–2022). Certain elements that are now essential to optogenetics were identified in earlier forms and different contexts as far back as 50 years ago. However, they were not conceptualized or developed as a means of controlling cellular function. In 1971, Oesterhelt D described bacteriorhodopsin as a light-activated ion pump (Oesterhelt and Stoeckenius, 1971). This discovery laid the foundation for the original theme of optogenetics that continued with the identification of other members of this protein family, including halorhodopsin in Matsuno-Yagi and Mukohata (1977), and channelrhodopsin (ChR) in Nagel et al. (2002). It was not until the 21st century that scientists successfully explored the precise control of neuron activity using these proteins. In 2005, Deisseroth K (Boyden et al., 2005) first reported that Channelrhodopsin-2, a rapidly gated light-sensitive cation channel, allows for the use of light to alter neural processing at the level of single spikes and synaptic events; by 2010, multiple opsins, such as channelrhodopsin, bacteriorhodopsin, and halorhodopsin, had demonstrated their ability to rapidly and safely activate or inhibit neurons, paving the way for a new era in optogenetics. This breakthrough opened up new possibilities for controlling and understanding neural circuits in intact tissues, leading to a wide range of applications in neuroscience and beyond. The bibliometric analysis results show an explosive growth of optogenetics-related papers after 2010, and the number of publications reached nearly a thousand in 2020, suggesting that optogenetics is a rapidly evolving and exciting field of research. Many optogenetics-related papers have been published in high-quality journals, such as NATURE, SCIENCE, and CELL, and have received significant attention from the scientific community. These papers have advanced our understanding of neural information processing and revolutionized the way we explore and treat neurological and psychiatric disorders. Moreover, these papers regarding optogenetics often have high citation rates, indicating their importance and impact on the scientific community. Some authors, such as Deisseroth K, Boyden ES, and Hegemann P et al., have become leading figures in the field, and their contributions have inspired and influenced many researchers worldwide. Optogenetics encompasses a wide range of fields, including neurosciences, molecular biology, neuroimaging, and materials science, where it contributes its value in distinct ways. With continued advances in optogenetics technology and applications, the field is expected to grow and contribute to our understanding of physiological and pathological processes.

### 4.1. Optogenetic components and techniques

Optogenetic components and techniques, optogenetics and neural circuitry, and optogenetics and disease were three clusters identified by co-occurring analysis. The wide-ranging impact of optogenetics is inseparable from the advancement of optogenetic techniques. Opsins is one of the three elements of optogenetics. The thematic map indicates that opsins is a hot keyword, while trend topic analysis shows that protein engineering has been a frequent topic in recent years. The family size of microbial rhodopsins, including ion pump, ion channel, and signaling/enzyme rhodopsins, has been expanding. Channelrhodopsin-2 (ChR2) is the first opsin successfully employed in freely moving mammals, followed by inhibitory halorhodopsin from *Natronomonas pharaonis* (NpHR). Various rhodopsins that possess specific or enhanced properties have been discovered or developed through engineering and genomic modifications in the past decade, such as highly expressing opsins (Berndt et al., 2011; Mattis et al., 2011; Yizhar et al., 2011), faster kinetics opsins for high-frequency excitation (Lin et al., 2009; Gunaydin et al., 2010; Klapoetke et al., 2014), spectrally shifted opsins for deep tissue projection (Zhang et al., 2008; Lin et al., 2013; Urmann et al., 2017), step-function opsins for chronic neuromodulation (Yizhar et al., 2011; Berndt et al., 2016), opsins with altered ion selectivity for inhibition (Berndt et al., 2014; Wietek et al., 2014; Govorunova et al., 2017, 2018, 2020, 2022), bidirectional regulation opsins for both activation and inhibition (Carus-Cadavieco et al., 2017; Vierock et al., 2021), and opsins for biochemical control (Airan et al., 2009; Yoshida et al., 2017; Henss et al., 2022). In earlier years, the discovery and study of opsins were in line with the development of optogenetics. This, in turn, resulted in a reverse translation of basic science, where optogenetics provided a new driving force for the study of microbial opsins. There is still a great demand for developing photosensitive opsins with improved properties to achieve cell regulation under different circumstances, leading to a sustained research interest in this area.

Optical neural interface or light delivery approach, another component of optogenetics, is also a hot theme according to our analysis. Previously, for optogenetic stimulation in cultured neuronal and acute slice preparations, an arc lamp could be used to deliver light through the imaging objective, allowing for full-field stimulation (Zhang et al., 2007). In 2007, the first instance of optical manipulation of behavior in freely moving rodents was shown through an intracranial optical fiber that was directly connected to a laser-diode light source (Adamantidis et al., 2007). Further research has improved upon this method, enabling long-term, stable placement of the optical fiber for many behavioral studies (Zhang et al., 2010). The delivery of light through fiber has mainly been achieved using laser sources or light-emitting diodes, with each method having its advantages. The drawbacks of optogenetics with optical fibers include the difficulty of precise surgery, increased risk of infection, irreversible tissue damage, and limitations on some behavioral activities due to attendant equipment. Various devices have emerged aiming to achieve wireless control (Wentz et al., 2011; Kim et al., 2013; Kathe et al., 2022), which is a frequent keyword in recent years according to the trend topic analysis. Despite concerns about output power, future technological advancements in wireless optogenetics could prove useful, especially for handling-sensitive animals, as well as experiments that cannot easily accommodate wire couplings. Recently, some interdisciplinary studies have combined nanomaterials with optogenetics, providing new ideas for research on wireless optogenetics (Chen et al., 2018; Hong, 2020). In the years ahead, there will be exciting opportunities for developing novel advanced optical neural interfaces. Moreover, there is growing interest in clinically inspired devices, such as optical cuff for optogenetic control of the peripheral nervous system, which are currently being tested in animal models (Michoud et al., 2018; Song et al., 2018; Zhang et al., 2019).

Similar to other gene therapies, optogenetics relies on viral vectors to deliver opsins to specific cells, with adeno-associated viruses (AAV) being one of the most commonly utilized viral tools in both basic research and clinical trials. However, there are still significant challenges that need to be addressed, including potential immune reactions, transduction specificity, clearance by the liver, and packaging capacity. Therefore, developing AAVs with novel features has become an important theme in optogenetics research in recent years. Capsid engineering, *in vivo* selection, and directed evolution offer promising avenues for improving AAV vectors. To increase the efficiency and feasibility of targeting cells using AAV, strategies such as developing isolates or serotypes with low immunogenicity or modifying the gene sequence of the antigenic part of AAV are being pursued (Zhang et al., 2022). The use of AAVs as a delivery vehicle may be limited due to their size restriction. Trans-splicing is a recently developed approach to increase the capacity of AAVs, achieved by splitting the gene of interest and packaging its two portions in separate vectors, which are then co-infected into the same cell to form concatemers and express the transgene as a single gene (Colella et al., 2018; Tornabene et al., 2019; Tornabene and Trapani, 2020). In addition, the administration of AAVs to target cells is challenging, with current stereotactic techniques carrying infection risks and the potential for injury, as well as limited accuracy. Peripheral delivery may result in liver accumulation and poor target organ specificity. Several AAV serotypes, including PHP.B (Deverman et al., 2016) and PHP.S (Challis et al., 2019) or AAV capsid variants (Goertsen et al., 2022) targeting the central nervous system, have been developed. Another option for brain delivery could involve using focused ultrasound to disrupt the blood–brain barrier (Chen et al., 2019). Besides AAV serotypes, one alternative method to achieve specificity for a particular type of cell is using eukaryotic promoters. However, there is still a need for further advancements to attain a high degree of cell specificity. Improving the performance of AAV is likely to remain a crucial and ongoing hot topic in the future.

Optogenetics technology required the development and maturation of three distinct technical aspects: microbial opsins, *in vivo* optics, and targeted genetic expression. The broad application of optogenetics did not occur until 2010, as its implementation was challenging and required collaborative efforts from many scientists and laboratories across various fields. Scientists have made sustained efforts, and even now, the continued improvement of the relevant components remains a hot and critical issue.

### 4.2. Optogenetics and neural circuitry

Optogenetic methods have revolutionized the field of neuroscience, shedding light on how specific cell types and neural projections play a causal role in both normal physiological processes and disease-related behaviors, such as memory, sense, pain, cognition, stress, vision, action, addiction, et al. (Zhang et al., 2022). Optogenetics is inherently characterized by high resolution in space and time, providing opportunities for novel experiments aimed at dissecting the function of specific neural patterns, which is not achievable by traditional methods. These studies involve analyzing circuit connectivity, discriminating cell subtypes and exploring cell functions, monitoring the dynamic signals transmission, or generating brain-wide activity maps integrated with other imaging techniques such as fMRI or PET (Lee et al., 2010). Initially, optogenetic research focused primarily on brain or spinal cord neurons. However, the scope of research has gradually expanded to include the peripheral nervous system and non-neuronal systems, such as skeletal, smooth or cardiac muscles, glial, stem cells, endocrine cells, et al. (Jia et al., 2011; Bruegmann et al., 2015; Park et al., 2017; Xie et al., 2020; Asano et al., 2021; Yang et al., 2022). Optogenetics has also led to many new discoveries about the neural circuitry underlying disease-related symptoms. The exact mechanisms at the circuit level of neuropsychiatric diseases have been elusive. Optogenetic studies have provided insights into the normal functioning of neural circuits and how they are disrupted in disease states, such as epilepsy, Parkinson’s disease, Alzheimer’s disease, Huntington’s disease, et al. (Roy et al., 2016; Cela and Sjostrom, 2019; Barry et al., 2020; Fougère et al., 2021). The knowledge can then be applied to better understand and treat neurological and psychiatric disorders. Optogenetics owes its existence to the unique feature of high precision and has since become a prominent and captivating research method. As a result, using optogenetics to explore neural circuits and projections has remained a trending topic.

### 4.3. Optogenetics and disease intervention

Despite challenges, ongoing research aimed at exploring the potential of optogenetics for clinical applications, which has been a burning issue recently. Research has explored the feasibility of ophthalmic optogenetics in animal experiments (Chuong et al., 2014; Sengupta et al., 2016). The area of vision restoration is the first and only field that has entered clinical trials (NCT05294978, NCT04945772, NCT05417126, NCT02556736, NCT04919473, NCT03326336). Sahel et al. (2021) reported the first clinical case of partial recovery of visual function in a neurodegenerative disease through intraocular injection AAV encoding ChrimsonR with light stimulation via engineered goggles. There is also hope that optogenetics can assist in restoring hearing for patients. Researchers have designed optical cochlear implants (oCIs), which convert the sound signal into an optical signal, thereby replacing traditional artificial cochlear implants (Klein et al., 2018; Dieter et al., 2020; Keppeler et al., 2020; Bali et al., 2021; Zerche et al., 2023). Currently, the development of oCIs is still in its early stages, and scientists are striving to improve its frequency resolution and sensitivity. Deep brain stimulation (DBS), which involves surgically implanted electrodes to deliver electrical stimulation to a specific brain region, has been approved as a therapeutic intervention for Parkinson’s disease. There is research aimed at using opto-DBS to target specific cell types or brain regions to address the non-specificity issue of traditional DBS (Chen et al., 2015; Gittis and Yttri, 2018). The treatment of Alzheimer’s disease (Roy et al., 2016; Etter et al., 2019) and epilepsy (Bentley et al., 2013) holds great promise as potential application areas. However, current optogenetic techniques are not yet sufficiently mature to target the entire brain. There are currently no effective methods for simultaneously integrating viral sequences into the host genome throughout the brain, urging the design of a novel optogenetic solution. Studies in preclinical models have successfully explored optogenetic treatments for pain relief, but the application in humans is still a long way off (Bonin et al., 2016; Wang et al., 2016). The potential of optogenetics has also been explored in cardiac defibrillation (Bingen et al., 2014; Majumder et al., 2020), bladder regulation (Mickle et al., 2019), and muscle paralysis (Williams et al., 2019). Based on the bibliometric analysis, we found that although clinical applications are still in their infancy, optogenetics is a significant trend for the future and deserves further attention.

## 5. Conclusion

This study investigated the development patterns, frontiers, and research hotspots in optogenetics on a global scale. The number of publications related to optogenetics has been on the rise since 2010, suggesting that this field of study is growing in importance. Using bibliometric analysis, we identified the main areas of research interest, which include optogenetic components and techniques, optogenetics and neural circuitry, and optogenetics and disease. Our results provide an overview of the current state and future research directions of optogenetics research.

## Data availability statement

The raw data supporting the conclusions of this article will be made available by the authors, without undue reservation.

## Author contributions

ZZ, XW, and XL were responsible for collecting data. ZZ was responsible for interpreting results and writing the manuscript. LL contributed to the study design and the editing of the manuscript. All authors contributed to the article and approved the submitted version.

## Funding

This study was funded by the Natural Science Foundation of Beijing, China (Grant no. 7222234), the fundamental research funds for central public welfare research institutes (Grant no. 2023CZ-1), the National Natural Science Foundation of China (Grant no. 82170792), and the Research Projects of China Rehabilitation Research Center (Grant no. 2021zx-10). The funders had no role in study design, data collection and analysis, decision to publish, or preparation of the manuscript.

## Conflict of interest

The authors declare that the research was conducted in the absence of any commercial or financial relationships that could be construed as a potential conflict of interest.

## Publisher’s note

All claims expressed in this article are solely those of the authors and do not necessarily represent those of their affiliated organizations, or those of the publisher, the editors and the reviewers. Any product that may be evaluated in this article, or claim that may be made by its manufacturer, is not guaranteed or endorsed by the publisher.
