# Corrigendum: A bibliometric profile of optogenetics: quantitative and qualitative analyses

**DOI:** 10.3389/fnins.2023.1258856

**Published:** 2023-07-27

**Authors:** Zhonghan Zhou, Xuesheng Wang, Xunhua Li, Limin Liao

**Affiliations:** ^1^Shandong University, Jinan, Shandong, China; ^2^Department of Urology, China Rehabilitation Research Center, Beijing, China; ^3^University of Health and Rehabilitation Sciences, Qingdao, Shandong, China; ^4^China Rehabilitation Science Institute, Beijing, China; ^5^Beijing Key Laboratory of Neural Injury and Rehabilitation, Beijing, China; ^6^School of Rehabilitation, Capital Medical University, Beijing, China

**Keywords:** optogenetics, bibliometrics, quantitative analysis, qualitative analysis, hot topic

In the published article, there was an error regarding the affiliations for Limin Liao. As well as having affiliations 2,3,4,5,6, they should also have affiliation ^1^ Shandong University, Jinan, Shandong, China.

The authors apologize for this error and state that this does not change the scientific conclusions of the article in any way. The original article has been updated.

